# Interactive effects of intrinsic capacity and obesity on the KDIGO chronic kidney disease risk classification in older patients with type 2 diabetes mellitus

**DOI:** 10.1186/s13098-022-00975-x

**Published:** 2023-01-02

**Authors:** Wei-Hua Tang, Teng-Hung Yu, Hui-Lan Lee, Yau-Jiunn Lee

**Affiliations:** 1 Division of Cardiology, Department of Internal Medicine, Taipei Veterans General Hospital, Yuli Branch, Hualien, 98142 Taiwan; 2 Faculty of Medicine, School of Medicine, National Yang Ming Chiao Tung University, Taipei, 112304 Taiwan; 3 Division of Cardiology, Department of Internal Medicine, E-Da Hospital, Kaohsiung, 82445 Taiwan; 4 School of Medicine, College of Medicine, I-Shou University, Kaohsiung, 82445 Taiwan; 5 Health Promotion Administration, Ministry of Health and Welfare, Taipei, 10320 Taiwan; 6 Lee’s Endocrinologic Clinic, No. 130 Min-Tzu Rd, Pingtung, 90000 Taiwan

**Keywords:** KDIGO chronic kidney disease risk classification, Intrinsic capacity, Obesity, Synergistic interaction, Type 2 diabetes mellitus

## Abstract

**Background:**

Intrinsic capacity (IC) is a novel concept focusing on normal and healthy aging. The effect of IC on the risk of chronic kidney disease (CKD) according to KDIGO category in older type 2 diabetes mellitus (T2DM) patients has rarely been studied. We investigated whether a decline in IC is associated with the risk of CKD according to KDIGO 2012 categories.

**Methods:**

This is a cross-sectional study. The exposure variables (IC score and body mass index) and outcome variable (KDIGO categories of the risk of CKD) were collected at the same timepoint. A total of 2482 older subjects with T2DM managed through a disease care program were enrolled. The five domains of IC, namely locomotion, cognition, vitality, sensory, and psychological capacity were assessed. Based on these domains, the IC composite score was calculated. CKD risk was classified according to the KDIGO 2012 CKD definition. Univariate and multivariate analyses were used to assess the association between IC score and KDIGO categories of risk of CKD.

**Results:**

The KDIGO CKD risk category increased in parallel with IC score (p for trend < 0.0001). In multivariate analysis, compared to those with an IC score 0, the odds ratio of having a KDIGO moderately increased to very high risk category of CKD was 1.76 (1.31–2.37) times higher for those with an IC score of 2–5. Furthermore, an increased IC score was associated with a higher prevalence of moderate and severe obesity. Moreover, there was a synergistic interaction between IC score and obesity on the KDIGO moderately increased to very high risk category of CKD (synergy index = 1.683; 95% CI 0.630–3.628), and the proportion of the KDIGO moderately increased to very high risk category of CKD caused by this interaction was 25.6% (attributable proportion of interaction = 0.256).

**Conclusions:**

Our findings indicate that IC score may be closely related to the KDIGO moderately increased to very high risk category of CKD. In addition, there may be a synergistic interaction between IC score and obesity, and this synergistic interaction may increase the KDIGO CKD risk stage.

## Background

The number of older people worldwide is projected to increase to over 1.5 billion within 30 years [1]. The incidence of diabetes mellitus (DM) is also increasing globally with the exponential increase in obesity [2]. Aging is inevitable, and so how to maintain a healthy active lifestyle later in life has become an important issue, both for individuals and social-economic systems [3].

More than 40% of diabetic patients will develop diabetic kidney disease and renal impairment despite controlling serum glucose status and other risk factors such as blood pressure and metabolic syndrome [4, 5]. In the past decades, numerous risk factors have been associated with chronic kidney disease (CKD) progression, and they have been grouped into six categories: sociodemographic and economic, behavioral, genetic, cardiovascular, metabolic, and several novel acute kidney injury biomarkers [6]. Although many promising biomarkers for CKD diagnosis and progression have been identified, few have been implemented into routine clinical practice [7]. This is because the prevention of DM-related CKD is very complex, especially in older patients, and it involves many psychological, cognitive, and behavioral aspects in addition to blood pressure and glucose control [8–10].

Intrinsic capacity (IC), defined as the composite of an individual’s mental and physical capacities and their interactions with relevant environmental characteristics, was introduced by the World Health Organization in 2015 [11]. IC measures the capacity of human biological and physiological systems, based on body and physical function [12]. Over time, IC may provide contextualized monitoring measurements which can then be used to inform clinical and public health policy [13]. Recent studies on IC have mainly focused on aging-related diseases, such as dementia [14, 15], and the use of IC in other diseases is rare. As impaired renal function always develops in older diabetic patients, the aim of this study was to evaluate the interactive effect of IC score and obesity on the KDIGO CKD risk classification among 2482 diabetic patients older than 65 years of age. To the best of our knowledge, this is the first study to apply the concept of IC to the KDIGO CKD risk classification in older DM patients.

## Materials and methods

### Study design and participants

Totally eight diabetes-specific local clinics in Southern Taiwan and the diabetic department of Kaohsiung E-Da Hospital, Taiwan jointed in this cross-sectional study. The exposure variables (IC score and body mass index (BMI)) and outcome variable (KDIGO categories of the risk of CKD) were collected at the same timepoint. A Total of 2482 older outpatient subjects with T2DM were enrolled. The inclusion criteria aged ≥ 65 years, clinically diagnosed with T2DM between January 2006 and October 2021, relatively healthy without acute illness and without evidence suggesting the possibility of a non-diabetic renal disease (included primary glomerular diseases, drug-induced nephropathy, reflux nephropathy, nephrolithiasis, polycystic kidney and renal-related infectious diseases). The exclusion criteria were patients: (1) aged < 65 years; (2) with type 1 diabetes; (3) with history with cancer, liver or urologic diseases; (4) who had been hospitalized for any reasons within 3 months prior to enrollment; (5) with recently use of allopurinol or uricosuric agents for gouty arthritis; (6) who underwent contrast examinations during the follow-up period; and (7) who could not provide complete demographics and personal medical information. In addition, to avoid the potential development/presence of primary glomerular diseases, we also excluded patients with persistent hematuria with and without urinary casts.

The diagnosis of T2DM was based on the World Health Organization criteria [16]. All of the patients were followed up in accordance with the diabetes comprehensive management program suggested by the Taiwan National Health Insurance at 3-month intervals. At each follow-up visit, standardized physical examinations, biochemical measurements after fasting, measurements of urine albumin and creatinine were performed. All participants received standard treatment based on recent updated diabetes, hypertension, and dyslipidemia management guidelines. All of the participants signed free and informed consent forms before enrollment into the study. Our study protocol and procedures were approved by E-Da Hospital Institutional Review Board with certificate number EMRP-108-111 and EMRP-109-109 and the Ethics Committees of Pingtung Christian Hospital with an approval certificate on 16 December 2005.

### Key measures

IC was determined using the ICOPE (WHO) screening tools, including six functional assessments of the following five domains: locomotion, cognition, vitality, sensory (visual and sensory), and psychological symptoms [17]. If subject was unable to complete five chair rises within 14 s, limited locomotion mobility was defined. If the patients gave an inappropriate answer to either of two questions on orientation in time and space, or could not recall the three words they were asked to remember, impaired cognitive dysfunction was defined. If subject suffered from weight loss greater than 3 kg over 3 months or with the loss of appetite, malnutrition was defined. If subject had any eye problems such as difficulty in seeing far, reading, eye diseases, or current ophthalmic medical treatment, a visual impairment was defined. If subject failed to hear whispers in the whisper test, a hearing loss was defined. If subject bothered by feeling down, feeling depressed or hopeless, or having little interest or pleasure in doing things over the previous 2 weeks, a depressive symptom was suggested. Finally, impairment in each item was scored as one point, and IC score was defined as the sum of the six functional assessments, with a higher score indicating greater functional impairment.

Obesity was defined according to the Ministry of Health and Welfare, Taiwan, criteria instead of the WHO criteria, as it has been suggested that the WHO BMI cut-off point for obesity (≥ 30 kg/m^2^) may be too high for Asians, thereby underestimating associated health risks [18, 19]. Accordingly, we defined underweight as BMI below 18.5 kg/m^2^, normal weight as between 18.5 ≤ BMI < 24 kg/m^2^, overweight as 24 ≤ BMI < 27 kg/m^2^, mild obesity as 27 ≤ BMI < 30 kg/m^2^, moderate obesity as 30 ≤ BMI < 35 kg/m^2^, and severe obesity as BMI greater than 35 kg/m^2^ [20].

Renal function (estimated glomerular filtration rate (eGFR)) was estimated using the CKD-EPI two-concentration race equation [21]: GFR = 141 × min(S_cr_ /κ, 1)^α^ × max(S_cr_ /κ, 1)^− 1.209^ × 0.993^Age^ × 1.018 [if female] × 1.159 [if black], where S_cr_ is serum creatinine (mg/dL), κ is 0.7 if females and 0.9 if males, α is − 0.329 if females and − 0.411 if males, min indicates the minimum of S_cr_/κ or 1, and max indicates the maximum of S_cr_/κ or 1. Albuminuria was defined by the albumin-to-creatinine ratio (UACR) from spot urine. The presence of albuminuria was defined by at least two measurements of UACR > 30 mg/g in a 6-month period during follow-up. The outcome was the classification of the patients in 4 categories based on their risk for CKD according to KDIGO 2012 using a combination of eGFR and albuminuria as follows: low risk group (eGFR ≥ 60 mL/min/1.73 m^2^ and UACR < 30 mg/g), moderately increased risk group (eGFR > 60 mL/min/1.73 m^2^ and 30 < UACR < 300 mg/g, or 45 < eGFR < 60 mL/min/1.73 m^2^ and 30 < UACR < 300 mg/g), high risk group (30 < eGFR < 60 mL/min/1.73 m^2^ and UACR > 300 mg/g, or eGFR > 60 mL/min/1.73 m^2^ and UACR > 300 mg/g), and very high risk group (15 < eGFR < 60 mL/min/1.73 m^2^ and UACR > 300 mg/g, or eGFR < 15 mL/min/1.73 m^2^ and UACR > 300 mg/g) [22].

### Laboratory measurements

Routine tests including a clinical examination, recent medication side effect assessment, body weight, blood pressure, urinary sediment and urinalysis, complete blood count, serum chemistry, and HbA1c concentrations were performed during each regular visits. The urinary albumin concentration was measured after overnight fasting by immunoturbidimetry (Beckman Instruments, Galway, Ireland). The detection limit was 2 mg/L, with the interassay and intraassay coefficients of variance < 8%. In the study admission, the patients were defined as normoalbuminuric if they had a UACR < 30 mg/g in at least two consecutive overnight urine collections. If the patient had first UACR measurement > 30 mg/g, a repeat urine test will be asked and checked to confirm the diagnosis of albuminuria within 3–6 months in the follow-up period later. If the urine specimen showed the presence of urinary infections, the specimen will not be used and a new sample was collected after antibiotics treatment. To exclude primary renal diseases, abnormal urinary sediment should not be noted in the urine specimen (presence of any protein, red blood cells, hemoglobin, white blood cells, nitrites or casts). Serum creatinine was measured by the Jaffe method. Serum HbA1C, total cholesterol, high-density lipoprotein cholesterol (HDL-C), low-density lipoprotein cholesterol (LDL-C), triglycerides, hemoglobin, creatinine, and glucose were determined using a parallel-multichannel analyzer (Hitachi 7170A, Tokyo, Japan) by standard commercial methods after an overnight fast as in our previous report [23].

### Variables

All participants completed a standard questionnaire that assessed age, gender, cigarette use, history of disease (T2DM, diabetes duration, hyperlipidemia, hypertension, heart disease, and cancer) in face-to-face interviews with trained interviewers. Subject’s blood pressure was measured by trained clinical assistants with digital automatic blood pressure monitor (model HEM-907; Omron, Omron, Japan) after resting for 5 min. Hypertension was defined as a systolic blood pressure (SBP) ≥ 140 mmHg, a diastolic blood pressure (DBP) ≥ 90 mmHg, or if the patient was recent using antihypertensive medication. Anthropometric parameters including BMI (kg/m^2^) were measured. Hyperlipidemia was defined according to the ATP III criteria as following: triglycerides ≥ 150 mg/dl, and/or HDL-C < 35 mg/dl in men or < 39 mg/dl in women, and/or total cholesterol ≥ 200 mg/dl, and/or LDL-C ≥ 130 mg/dl, or those undergoing treatment for lipid disorders.

### Statistical analysis

Data normality was analyzed using the Kolmogorov–Smirnov test. Continuous, normally distributed variables are presented as mean ± SD, and non-normally distributed variables as median (interquartile range). Categorical variables are presented as frequencies and/or percentages. Baseline characteristics were compared between groups using one-way analysis of variance (ANOVA) for normally distributed variables. The chi-square test was used to compare categorical variables.

Logistic regression models were used to estimate odds ratios (ORs) and 95% confidence intervals (CIs) for the risk of CKD in each IC score, compared with an IC score of 0 as the reference. To test linear risk trends, a tertiles as a continuous variable in the regression models was used.

ORs and corresponding 95% CIs were calculated using univariate and multivariate logistic regression models to evaluate the relationships between IC scores and the risk of CKD. A *p* value < 0.05 was considered to be statistically significant. JMP version 7.0 for Windows (SAS Institute, Cary, NC, USA) was used in our analysis.

An Excel sheet provided by Andersson and co-authors [24] was used into the database and compute the relevant indicators of interactions. Using a logistic regression model, a value was obtained and taken as the estimated additive interaction between IC score and obesity status. The interaction based on the additive model was determined using the following indexes: the relative excess risk of interaction (RERI), attributable proportion of interaction (API), synergy index (SI), measure of multiplicative interaction for risk ratios [25] and their 95% CIs using the delta method [26]. The RERI refers to the excess risk due to the interaction relative to the risk without exposure. The API is the attributable proportion of disease caused by the interaction in subjects with both exposures. The SI refers to the excess risk from both exposures when there is a biological interaction due to the risk from both exposures without interaction. The RERI has been showed to be the best measure of interaction using a proportional hazards model [27]. If the RERI and AP are equal to 0, absence of additive interactions was defined [28]. Finally, an indicative biological interaction is considered when RERI > 0, AP > 0, S > 1, or a measure of multiplicative interaction for risk ratios > 1.

## Results

### Characteristics of the participants

A total of 2482 patients with T2DM aged 65–99 years were included, and their baseline characteristics and clinical data are presented in Table 1. The mean ± SD age was 72.4 ± 5.8 years and known duration of diabetes was 13.5 ± 9.0 years. The prevalence rates of hypertension, hyperlipidemia, and smoking were 62.5%, 78.8%, and 15.6%, respectively. The patient’s mean eGFR was 68.9 ± 22.0 mL/min/1.73 m^2^, and the median (interquartile range) UACR was 19.2 mg/g (9.7–54.3 mg/g).

**Table 1:** Main characteristics according to intrinsic capacity score

	Total	IC score 0	IC score 1	IC score ≥ 2	p-value
Number	2482	1525	655	302	
Age (years)	72.4 ± 5.8	71.2 ± 5.0	73.2 ± 5.9	77.0 ± 6.6	< 0.0001
Sex, female (n, %)	1374(55.4)	790(51.8)	393(60.0)	191(63.3)	< 0.0001
Diabetes duration (years)	13.5 ± 9.0	13.1 ± 8.8	13.7 ± 9.2	15.2 ± 9.2	0.001
Hypertension (n, %)	1552(62.5)	917(60.1)	438(66.9)	197(65.2)	0.007
Hyperlipidemia (n, %)	1956(78.8)	1217(79.8)	503(76.8)	236(78.2)	0.276
Current smoker (n, %)	486(19.6)	315(20.7)	121(18.5)	50(16.6)	0.184
Body mass index (kg/m^2^)	25.6 ± 4.0	25.4 ± 3.8	26.0 ± 4.2	25.8 ± 4.2	0.001
Waist-to-hip ratio	0.93 ± 0.07	0.92 ± 0.07	0.93 ± 0.07	0.93 ± 0.07	0.156
SBP (mmHg)	129 ± 16	128 ± 15	130 ± 16	130 ± 19	0.076
DBP (mmHg)	73 ± 11	73 ± 11	73 ± 11	73 ± 11	0.669
Obesity status (n, %)					
Underweight	35(1.4)	19(1.3)	11(1.7)	5(1.7)	0.681
Normal weight	886(35.7)	571(37.4)	210(32.1)	105(34.8)	0.052
Overweight	782(31.5)	485(31.8)	199(30.4)	98(32.5)	0.752
Mild obesity	451(18.2)	278(18.2)	129(19.7)	44(14.6)	0.160
Moderate obesity	274(11.0)	147(9.6)	88(13.4)	39(12.9)	0.019
Severe obesity	54(2.2)	25(1.6)	18(2.8)	11(3.6)	0.047
KDIGO CKD risk stage (n, %)					
Low risk	1167(47.0)	789(51.7)	289(44.1)	89(29.5)	< 0.0001
Moderately increased risk	610(24.6)	379(24.9)	154(23.5)	77(25.5)	0.740
High risk	368(14.8)	192(12.6)	108(16.5)	68(22.5)	< 0.0001
Very high risk	337(13.6)	165(10.8)	104(15.9)	68(22.5)	< 0.0001
Type of treatment (%)					
(OHA/Insulin/Both)	67.8/3.2/29.1	71.6/2.1/26.3	65.5/4.6/30.0	53.7/5.3/41.0	< 0.0001
Statins (n, %)	1919(77.3)	1193(78.2)	494(75.4)	232(76.8)	0.348
ARB (n, %)	1085(43.7)	626(41.1)	317(48.4)	142(47.0)	0.003
Biochemical characteristics					
HbA1c (%)	7.3 ± 1.2	7.2 ± 1.1	7.2 ± 1.2	7.5 ± 1.4	0.006
Fasting glucose (mmol/L)	8.5 ± 3.4	8.4 ± 3.3	8.5 ± 3.4	8.9 ± 3.8	0.001
Total cholesterol (mmol/L)	4.1 ± 0.8	4.1 ± 0.8	4.1 ± 0.8	4.0 ± 0.8	0.671
Triglycerides (mmol/L)	1.3 ± 0.7	1.3 ± 0.8	1.3 ± 0.7	1.3 ± 0.6	0.694
HDL cholesterol (mmol/L)	1.4 ± 0.4	1.4 ± 0.4	1.4 ± 0.4	1.4 ± 0.4	0.986
LDL cholesterol (mmol/L)	2.1 ± 0.6	2.1 ± 0.6	2.1 ± 0.7	2.1 ± 0.6	0.503
Uric acid (mmol/L)	5.4 ± 1.6	0.3 ± 0.1	0.3 ± 0.1	0.3 ± 0.1	0.841
eGFR (ml/min/1.73m^2^)	68.9 ± 22.0	77.2 ± 20.5	73.3 ± 21.5	71.0 ± 23.0	< 0.0001
UACR (mg/g)	19.2(9.7–54.3)	17.1(8.8–46.5)	21.0(10.9–60.9)	29.3(13.4–83.6)	< 0.0001
Creatinine (µmol/L)	97.2 ± 53.0	94.6 ± 48.6	102.5 ± 67.2	107.9 ± 56.6	< 0.0001
Hemoglobin (g/L)	136 ± 17	138 ± 17	133 ± 17	131 ± 17	< 0.0001

Data are presented as mean ± SD, number (percentage), or median (interquartile range)

*IC* intrinsic capacity, *SBP* systolic blood pressure, *DBP* diastolic blood pressure, *CKD* chronic kidney disease, *OHA* oral hypoglycemic agents, *ARB* angiotensin receptor blocker, *HDL* high-density lipoprotein, *LDL* low-density lipoprotein, *eGFR* estimated glomerular filtration rate, *UACR* urinary albumin-to-creatinine ratio

### Main characteristics according to IC score

Furthermore, the general characteristics of the 2482 patients grouped according to IC scores are also reported in Table 1. The numbers of patients with an IC score of 0, 1, and 2–5 were 1525 (61.4%), 655 (26.4%), and 302 (12.2%), respectively. The patients with an IC score 2–5 were more predominantly female, had severe obesity, higher prevalence of KDIGO high risk and very high risk categories of CKD, higher rates of both insulin and oral hypoglycemic agents, older age, longer diabetes duration, higher HbA1c, fasting glucose, UACR, and creatinine levels, and lower prevalence of KDIGO low risk category of CKD, eGFR, and hemoglobin levels than those with an IC score of 0 or 1. Moreover, the patients with an IC score 2–5 had a higher prevalence of hypertension and moderate obesity, and higher rate of treatment with angiotensin receptor blockers than those with an IC score of 0. There were no significant differences in hyperlipidemia, current smoker, waist-to-hip ratio, SBP, DBP, underweight, normal weight, overweight, mild obesity, KDIGO moderately increased risk category of CKD, statin treatment, total cholesterol, triglycerides, HDL cholesterol, LDL cholesterol, and uric acid among the three groups.

### Association between IC score and KDIGO moderately increased risk to very high risk category of CKD

We investigated associations between IC score and KDIGO moderately increased risk to very high risk category of CKD (Table 2). The KDIGO moderately increased risk to very high risk category of CKD increased in parallel with IC score. Accordingly, there were increases in the ORs for the association with KDIGO moderately increased risk to very high risk category of CKD relative to an IC score of 0, OR = 1.0; score 1, OR = 1.36; score 2–5, OR = 2.57 (p for trend across increasing IC scores < 0.0001).

**Table 2:** Odds ratio (ORs) of KDIGO moderately increased risk to very high risk category of chronic kidney disease according to intrinsic capacity score

Variables	Chronic kidney disease risk stage^a^			p-value
	Moderate risk to very high risk group	Low risk group	OR (95%CI)	
IC score				
Score 0	736 (48.3%)	789 (51.7%)	1.00 (reference)	
Score 1	366 (55.9%)	289 (44.1%)	1.36 (1.13–1.63)	0.001
Score ≥ 2	213 (70.5%)	89 (29.5%)	2.57 (1.97–3.36)	< 0.0001
P for trend			< 0.0001	

*IC* intrinsic capacity, *OR* odds ratio, *CI* confidence interval

^a^CKD risk stage was defined according to the 2012 KDIGO definition [22]

### Association of IC score with KDIGO moderately increased risk to very high risk category of CKD

We used univariate and multivariate logistic regression models to investigate associations between IC score and KDIGO moderately increased risk to very high risk category of CKD (Table 3). Patients with an IC score of 1 had an increased KDIGO moderately increased risk to very high risk category of CKD compared with those who had an IC score of 0 in model 1 and model 2. However, those with an IC score of 1 did not have an increased KDIGO moderately increased risk to very high risk category of CKD compared with those who had an IC score of 0 in model 3. Patients with an IC score of 2–5 had a higher KDIGO moderately increased risk to very high risk category of CKD compared with those with an IC score 0 in model 1, model 2, and model 3 (OR: 2.57, 95% CI 1.97–3.36, p < 0.0001, OR:1.82, 95% CI 1.38–2.42, p < 0.0001, and OR: 1.76, 95% CI 1.31–2.37, p = 0.0002, respectively).

**Table 3:** Logistic regression of the association of intrinsic capacity score with KDIGO moderately increased risk to very high risk category of chronic kidney disease

	Model 1	Model 2	Model 3
Variables	OR (95%CI) p-value	OR (95%CI) p-value	OR (95%CI) p-value
IC score			
0	Ref	Ref	Ref
1	1.36 (1.13–1.63) 0.001	1.21 (1.00–1.46) 0.049	1.12 (0.92–1.37) 0.271
≥ 2	2.57 (1.97–3.36) < 0.0001	1.82 (1.38–2.42) < 0.0001	1.76 (1.31–2.37) 0.0002

*OR* odds ratio, *CI* confidence interval, *IC* intrinsic capacity

Model 1: Univariate logistic regression analysis

Model 2: Adjusted for age, gender

Model 3: Adjusted for age, gender, diabetes duration, smoking status, body mass index, systolic blood pressure, diastolic blood pressure, total cholesterol, high-density lipoprotein cholesterol, low-density lipoprotein cholesterol, triglycerides, fasting sugar

### Joint impacts of IC score and obesity on the KDIGO moderately increased risk to very high risk category of CKD

**Fig. 1:**
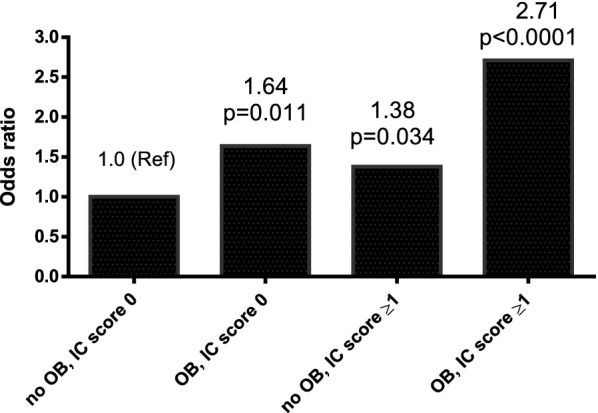
Interaction schematic diagram between moderate and severe obesity (OB) and intrinsic capacity (IC) score on KDIGO moderately increased risk to very high risk category of chronic kidney disease after adjusting for multiple confounders

Because an increased IC score was associated with higher moderate and severe obesity, we investigated the additive interaction effect of IC score and moderate and severe obesity on the KDIGO moderately increased risk to very high risk category of CKD, including IC score 0 and normal weight, IC score 0 and moderate and severe obesity, IC score 1–5 and normal weight, and IC score 1–5 and moderate and severe obesity. In univariate analysis, the patients with an IC score 0 and moderate and severe obesity had a 1.90-fold higher KDIGO moderately increased risk to very high risk category of CKD (OR = 1.90; 95% CI 1.35–2.69, p = 0.0003) than those without. In addition, the patients with an IC score 1–5 and normal weight had a 1.69-fold higher moderately increased risk to very high risk of KDIGO CKD risk category (OR = 1.69; 95% CI 1.29–2.23, p = 0.0002) than those without. Moreover, the patients with an IC score 1–5 and moderate and severe obesity had a 3.12-fold higher moderately increased risk to very high risk of KDIGO CKD risk category (OR = 3.12; 95% CI 2.14–4.60, p < 0.0001) than those without (data not shown). In multivariate analysis, the patients with an IC score 0 and moderate and severe obesity had a 1.64-fold higher moderately increased risk to very high risk of KDIGO CKD risk category (OR = 1.64; 95% CI 1.12–2.39, p = 0.011) than those without. Furthermore, the patients with an IC score 1–5 and normal weight had a 1.38-fold higher moderately increased risk to very high risk of KDIGO CKD risk category (OR = 1.38; 95% CI 1.03–1.86, p = 0.034) than those without. Moreover, the patients with an IC score 1–5 and moderate and severe obesity had a 2.71-fold higher moderately increased risk to very high risk of KDIGO CKD risk category (OR = 2.71; 95% CI 1.82–4.10, p < 0.0001) than those without (Fig. 1). The RERI, API, SI, and measure of multiplicative interaction for risk ratios were 0.695, 0.256, 1.683, and 1.200, respectively. The RERI > 0, API > 0, SI > 1, or a measure of multiplicative interaction for risk ratios > 1 suggested that there may be a synergistic interaction between IC score 1–5 and moderate and severe obesity on the risk of KDIGO moderately increased risk to very high risk category. In addition, the API was 0.256 after adjusting for all confounders, indicating that the proportion of risk of KDIGO moderately increased risk to very high CKD risk category that may have been caused by the interaction of IC score 1–5 and moderate and severe obesity was 25.6% in the patents with a KDIGO moderately increased risk to very high risk category of CKD.

## Discussion

In this study, we found that IC score was related to and had a synergistic interaction with obesity on the KDIGO moderately increased risk to very high risk category of CKD in diabetic patients aged ≥ 65 years. IC is a novel concept of healthy aging introduced by the WHO to help develop public health strategies in response to the aging population, as adults older than 60 years are expected to account for 12% to 22% of the global population (approximately 2 billion people) by 2050 (https://www.who.int/news-room/fact-sheets/detail/ageing-and-health). Most recent studies using IC score have focused on frailty, physical resilience and dementia, and rarely on other chronic diseases [17, 29]. Older populations have more comorbidities, which may impair renal function. In this study, we found that the IC score was very sensitive, as even a score of 1 was associated with an increased KDIGO CKD risk staging, and this association persistent after adjustments using logistic regression analysis (Table 3).

We found that IC score was associated with age, female sex, hypertension and obesity status, and a higher IC impairment score was associated with higher BMI, HbA1c, fasting glucose, UACR, creatinine levels, and the use of insulin and angiotensin receptor blockers, and lower eGFR and hemoglobin levels than those with an IC score of 0 or 1 (Table 1). Associations between IC score with demographic characteristics and metabolic control profiles have seldom been described, although a recent study reported that dyslipidemia and not hypertension status was associated with IC score [30, 31]. Most previous studies on IC have focused on interactions among chronic diseases, self-care capacity and social engagement, but not on disease progression or organ function. Two studies reported that impaired IC may be related to renal function and elevated heart failure markers [15, 32], and two other studies reported that impaired renal function was associated impaired mobility and poor nutrition [31, 33]. Therefore, it is important and reasonable to investigate and clarify the associations and interactions between IC score and the risk of renal function impairment, demographic characteristics and metabolic control profiles. The detailed pathogenetic mechanisms between IC and metabolic derangement are discussed below.

The most important finding of this study is the synergistic interaction of IC score and obesity on the KDIGO moderately increased risk to very high risk category of CKD after analyzing multiple interactions for risk ratios (Fig. 1). Several recent studies have reported that obesity is associated with older age and increased rates of frailty and mobility, and that this impairs the patient’s quality of life [34]. In 2000, Baumgartner termed the phrase sarcopenic obesity [35], and it has been shown to be especially prevalent and associated with many adverse health conditions in older adults [36]. Sarcopenic obesity is defined as the combination of obesity with low muscle mass and strength [35]. Although it can be caused by age-related changes in body composition [37], several pathways have also been proposed, including a sedentary lifestyle with less physically activity [38], higher inflammation status [39], insulin resistance [40], lower growth hormone and testosterone levels [41, 42], relative malnutrition [43], and poor psychological status [44], all of which occur in older patients.

In a literature review, sarcopenic obesity was associated with frailty among older adults [45], and with the risk of coronary artery disease and all-cause mortality [46, 47]. Recent studies have further shown an association between sarcopenic obesity and chronic renal disease, especially in those with diabetes-related renal impairment [48]. This may explain why insulin resistance, the over-expression of adipokines, and inflammation processes have been associated with obesity and CKD progression [49–53]. Taken together with frailty, it is reasonable that all of these adverse conditions could impair IC and aggravate the deterioration in renal function. Our study provides further information to support that obesity with pre-frailty is associated with the pathogenesis of CKD.

The interactions among muscle wasting, obesity and diabetic nephropathy are unclear. Advanced glycosylated end products (AGEs) have been associated with hyperglycemia, muscle wasting, and impaired renal function in vivo and in vitro [54], In addition, insulin resistance, oxidative stress, inflammation, uremic toxin toxicity, metabolic acidosis, vitamin D deficiency and protein energy wasting occur in patients with diabetic nephropathy, and they also result in muscle loss and abnormal fat deposition in CKD patients [48]. Taken together, these findings show that it is important to detect and evaluate sarcopenic obesity to allow for the more intensive management of diabetes and renal dysfunction in older patients.

Most of the current strategies to prevent the complications of diabetic nephropathy still focus on preventing hyperglycemia, the early diagnosis of kidney disease, and antihypertensive treatment to reduce renin-angiotensin system activity [48]. To reduce complications and disability directly or indirectly caused by diabetes, the American Diabetes Association has also launched numerous projects, such as the Diabetes Self-Management Education and Support (DSMES) and ADCES7 Self-Care Behaviors^™^ to help clinical physician and patients to monitor clinical, psychosocial and behavioral aspects of diabetes [55, 56]. However, none of these focus on the evaluation or detection of sarcopenic obesity-related CKD in diabetic patients.

Rapid and accessible assessment tools to evaluate the health and physical condition of older adults are needed, as not everyone can access medical facilities or afford the cost of biochemical and imaging examinations. Healthy aging is a process of developing and maintaining functional ability that enables well-being in old age. IC considers both physical and mental abilities by determining the functional ability combined with environmental factors and their interaction (https://apps.who.int/iris/handle/10665/ 186463), and it has been proven that using IC is more effective to assess older populations than focusing on specific diseases or biomarkers [15]. In this study, we applied the IC concept to older diabetic patients, and found that impaired IC combined with obesity could further screen out patients with KDIGO moderately increased risk to very high risk category of CKD even before changes in UCAR, eGFR and creatine levels, which are used to evaluate renal function and early CKD clinically.

There are still some limitations to this study. First, we lacked data on inflammation, adipocytokines and other specific kidney injury markers, and further investigations are warranted to investigate their association with IC and CKD progression. Second, due to case number limitation, although the impairment of IC with even an IC score of 1 showing a significant association with the KDIGO moderately increased risk to very high risk category of CKD, further evaluations are needed to clarify which components of IC impairment have the strongest effects. Third, the current observation is a preliminary cross-sectional association study, and further investigations are needed to examine the long-term predictive ability of IC score on the progression of CKD.

## Conclusions

This is the first study to show that IC impairment was associated with the KDIGO moderately increased risk to very high risk category of CKD, and that obesity status further interacted and synergistically increased the KDIGO categories of risk of CKD among older diabetic patients. Earlier interventions for IC impairment and obesity should be performed in this population to help prevent CKD.

## Data Availability

The datasets used and/or analysed during the current study are available from the corresponding author on reasonable request.

## Abbreviations

DM: Diabetes mellitus
CKD: Chronic kidney disease
IC: Intrinsic capacity
T2DM: Type 2 diabetes mellitus
BMI: Body mass index
eGFR: Estimated glomerular filtration rate
UACR: Urinary albumin-to-creatinine ratio
HDL-C: High-density lipoprotein cholesterol
LDL-C: Low-density lipoprotein cholesterol
SBP: Systolic blood pressure
DBP: Diastolic blood pressure
ANOVA: Analysis of variance
ORs: Odds ratios
Cis: Confidence intervals
RERI: Relative excess risk of interaction
API: Attributable proportion of interaction
SI: Synergy index
AGEs: Advanced glycosylated end products
DSMES: Diabetes self-management education and support
